# Bicarbonate-integrated transarterial chemoembolization (TACE) in real-world hepatocellular carcinoma

**DOI:** 10.1038/s41392-025-02400-x

**Published:** 2025-09-01

**Authors:** Kai Jin, Siying Zeng, Bin Li, Guangqiang Zhang, Jianjun Wu, Xun Hu, Ming Chao

**Affiliations:** 1 Interventional Radiology, The Second Affiliated Hospital, Zhejiang University School of Medicine, Hangzhou, China; 2 Cancer Institute (The Key Laboratory for Cancer Intervention and Prevention, China National Ministry of Education), The Second Affiliated Hospital, Zhejiang University School of Medicine, Hangzhou, China; 3 Department of Endocrinology, The Second Affiliated Hospital, Zhejiang University School of Medicine, Hangzhou, China; 4 Cancer Center of Zhejiang University, Hangzhou, Zhejiang China

**Keywords:** Cancer therapy, Gastrointestinal cancer

## Abstract

The objective response rate of conventional transarterial chemoembolization (TACE) for locoregional control of hepatocellular carcinoma (HCC) is approximately 50%. We previously developed bicarbonate-integrated TACE, termed TILA-TACE, which demonstrated 100% effectiveness for locoregional control of unresectable HCC. This study aimed to validate its efficacy, selectivity, and safety in real-world clinical practice (ChiCTR-ONC-17013416). A total of 413 patients were enrolled, including 40 (9.7%) with early-stage HCC, 29 (7.0%) with intermediate-stage HCC, and 344 (83.3%) with advanced-stage HCC. Primary tumors and macrovascular invasion/extrahepatic metastases were treated with TILA-TACE and radiation therapy, respectively. The side effects of TILA-TACE were recorded. The objective response rate of HCC tumors to TILA-TACE was 99.01%, including a complete response in 72.77% of patients. The objective response rate of tumor thrombus to radiation therapy was 96.88%. During a median follow-up of 38 months, there were 1 and 4 deaths among early- and intermediate-stage patients, respectively. The median survival of advanced-stage patients was 27 months. We found that intrahepatic metastases accounted for 70.4% (107/152) of cancer-related deaths after effective control of primary tumors and vascular invasion. The main adverse events associated with TILA-TACE were transient liver enzyme or bilirubin abnormalities (86.44% and 56.66%, respectively), which was consistent with the known side-effect profile of TACE. In conclusion, TILA-TACE is a novel and highly effective treatment for the local control of HCC with a tolerable safety profile. When combined with radiation therapy for macrovascular invasion, it offers significant survival benefits for patients with advanced HCC.

## Introduction

Hepatocellular carcinoma (HCC) is the sixth most common cancer and the third leading cause of cancer-related death worldwide, posing a significant global health burden, particularly in regions with high hepatitis B virus prevalence, such as East Asia.^1^ In China, the incidence and mortality of HCC remain particularly high. One of the major clinical challenges is that the majority of HCC patients are diagnosed at intermediate or advanced stages,^2,3^ when curative options such as surgical resection, radiofrequency ablation, or liver transplantation are no longer applicable due to extensive tumor burden, vascular invasion, or impaired liver function. For these patients, transarterial chemoembolization (TACE) remains the most commonly used locoregional therapy.

According to the Chinese Guidelines for the Diagnosis and Treatment of Primary Liver Cancer,^4,5^ conventional TACE (cTACE) is a recommended first-line option for patients with unresectable or intermediate-stage HCC. cTACE involves the intra-arterial administration of chemotherapeutic agents emulsified in lipiodol, followed by embolization of tumor-feeding arteries using agents such as gelatin sponge particles or microspheres. While cTACE has shown clinical benefits in many patients, its therapeutic efficacy varies widely. A comprehensive meta-analysis by Lencioni^6^ summarized TACE outcomes across studies from 1980 to 2013, reporting an average objective response rate (ORR) of 52.5% and a median overall survival (OS) of 19.4 months. More recent studies have reported similar ORRs values ranging from 19.5% to 58%,^7–10^ confirming the moderate and highly variable efficacy of cTACE in clinical practice.

This variability in efficacy is primarily attributed to the biological heterogeneity of HCC. Tumor size, vascularity, metabolic characteristics, and molecular features all influence treatment response. Several tumor-intrinsic factors have been associated with resistance to cTACE, including activation of the NRF2 antioxidant pathway,^11^ overexpression of pyruvate kinase M2 (PKM2),^12^ and activation of the HIF1α/pAKT signaling loop.^13^ These pathways promote tumor cell survival under stress conditions, such as hypoxia and nutrient deprivation induced by TACE. In addition, larger tumor size and the presence of hypovascular or infiltrative tumor components are associated with lower response rates.^14^

We previously hypothesize that intratumoral alkalization by bicarbonate may significantly improve therapeutic efficacy in TACE. This hypothesis is grounded in four lines of evidence:

First, the role of intratumoral lactic acidosis in tumor biology has been well-established. Pioneering work by the Gillies and Gatenby group^15^ demonstrated that systemic and tumor extracellular pH (pHe) alkalization suppressed carcinogenesis, invasion, and metastasis, while also developing predictive models for the safety and efficacy of buffer therapy.^16–18^ Subsequent theoretical studies further supported these findings.^19,20^

Second, we previously demonstrated that intratumoral lactic acidosis was a key resistance factor to nutrient deprivation.^21–23^ TACE blocks the arterial supply of oxygen and nutrients to the tumor, inducing ischemia and glucose starvation. However, lactic acidosis helps tumor cells adapt to glucose deprivation by maintaining metabolic homeostasis and suppressing cell death.^22,23^ We hypothesized that neutralizing lactic acidosis could disrupt this adaptive response and enhanced the cytotoxic effects of TACE.

Third, we previously demonstrated that alkalinization of pHi disrupts mitochondrial function by reducing the pH gradient (∆pH), membrane potential (∆Ψm), and proton motive force (∆p) across the inner mitochondrial membrane.^24^ This collapse of ∆p impairs oxidative phosphorylation (OXPHOS), autophagy, triggers sustained mitochondrial permeability transition (MPT), and leads to mitochondrial swelling, rupture, and ultimately cancer cell death.^24^

Fouth, sodium bicarbonate is the second most abundant inorganic ion in the human body and plays a critical role in buffering physiological pH. Bicarbonate is safe as long as the dosage of local injection into the tumor does not significantly affect the systemic pH.^18–20^

Based on these mechanistic insights, we developed a new protocol named TILA-TACE (targeting intratumoral lactic acidosis-TACE).^25^ In this protocol, 5% sodium bicarbonate is intermittently infused into the tumor via the feeding arteries in alternation with the doxorubicin-lipiodol emulsion, subjected to embolization. This approach aims to neutralize the intratumoral lactic acidosis and enhance the efficacy of chemoembolization. To evaluate this approach, we previously conducted both a retrospective controlled study and a randomized controlled trial (RCT), with sample sizes determined by rigorous power analysis. The results were striking: TILA-TACE achieved an ORR of 100% in both studies, compared to 44.4% in the cTACE group in the retrospective cohort and 63.6% in the RCT group. The geometric mean percentage of viable tumor residue was significantly reduced in the TILA-TACE group than in the cTACE group: in the retrospective cohort, 7.1% (TILA-TACE, n = 30) vs. 45.6% (cTACE, n = 27); in the RCT, 4.1% (TILA-TACE, n = 10) vs. 28.1% (cTACE, n = 10).

These results prompted us to further evaluate TILA-TACE in routine clinical settings. In the current study, we present a large, real-world cohort of 413 HCC patients treated with TILA-TACE across multiple disease stages. We aimed to assess the effectiveness, tumor selectivity, and safety of TILA-TACE in a heterogeneous patient population. This study builds on our prior work and provides important insights into the clinical applicability of TILA-TACE. We also report subgroup analyses of patients with advanced-stage disease, early-stage disease, and recurrent HCC, offering a comprehensive evaluation of this novel treatment approach.

## Results

### Baseline characteristics

Between November 2017 and December 2020, 413 patients with hepatocellular carcinoma (HCC) were enrolled and analyzed. The patients’ baseline characteristics are summarized in Table 1. The median age was 60 years (range, 18–89), with a male predominance (338/413, 81.84%). Hepatitis B virus (HBV) infection was the predominant etiology (349/413, 84.50%), and cirrhosis was present in 389 patients (94.19%). With respect to liver function, 365 patients (88.38%) were classified as Child‒Pugh class A, and 48 (11.62%) were classified as class B. Elevated alpha-fetoprotein (AFP) levels ( > 400 ng/mL) were observed in 151 patients (36.56%).

**Table 1:** Disease characteristics of patients

Variables	Patients	
	TILA-TACE	
**Patient number**	**413**	
First diagnosed	225	(54.48%)
Recurrent or metastatic HCC	188	(45.52%)
**Median age, years**	**60**	**(Range 18–89)**
**Sex (M/F)**	**338/75**	**(81.84%/18.16%)**
**Etiology**		
HBV	349	(84.50%)
HCV	0	(0%)
Non B-non C	64	(15.50%)
**Cirrhosis (radiology)**	**389**	**(94.19%)**
**Bilirubin, μM**	**18.07** ± **9.56**	
**Albumin, g/L**	**38.13** ± **4.71**	
**AST, U/L**	**55.85** ± **51.99**	
**ALT, U/L**	**42.14** ± **46.69**	
**AFP**		
≤20 ng/mL	161	(38.98%)
>20 ng/mL, ≤400 ng/mL	101	(24.46%)
>400 ng/mL	151	(36.56%)
**Child‒Pugh class, A/B**	**365/48**	**(88.38%/11.62%)**
**ECOG (score)**		
0	69	(16.71%)
1	344	(83.29%)
**The size of largest tumor: diameter cm (volume cm**^3^**)**	**0.5–20.5** **cm (0.07–4508.58** **cm**^3^**)**	
Tumor diameter>10 cm	76	(18.40%)
Tumor diameter>5 cm, ≤10 cm	144	(34.87%)
Tumor diameter ≤5 cm	193	(46.73%)
**CNLC stage**		
I	40	(9.69%)
II	29	(7.02%)
III^a^	344	(83.29%)
**Macrovascular invasion**	**270**	**(65.38%)**
Portal vein	149	(36.08%)
Hepatic vein	7	(1.69%)
Inferior vena cava	1	(0.24%)
Portal + hepatic vein	104	(25.18%)
Portal + inferior vena cava	4	(0.97%)
Hepatic + inferior vena cava	2	(0.48%)
Portal + hepatic + inferior vena cava	3	(0.73%)
***Portal vein invasion***	**260**	**(62.95%)**
*Invasion without PVTT*	136	(32.93%)
Involving subsegmental branches	48	(11.62%)
Involving segmental branches	55	(13.32%)
Involving the left or right branch	33	(7.99%)
Involving the main trunk	0	(0%)
*Invasion with PVTT*	124	(30.02%)
Involving subsegmental branches	3	(0.73%)
Involving segmental branches	27	(6.54%)
Involving the left or right branch	69	(16.71%)
Involving the main trunk	25	(6.05%)
***Hepatic vein invasion***	**116**	**(28.09%)**
Invasion without HVTT	97	(23.49%)
Invasion with HVTT	19	(4.60%)
***Inferior vena cava invasion***	**10**	**(2.42%)**
Invasion without IVCTT	3	(0.73%)
Invasion with IVCTT	7	(1.69%)
**Extrahepatic metastasis**	**60**	**(14.53%)**
Lung	21	(5.08%)
Lung + bone	4	(0.97%)
Lung + Lymph nodes	5	(1.21%)
Lung + Soft tissue	2	(0.48%)
Soft tissue	9	(2.18%)
Lymph nodes	9	(2.18%)
Bone	7	(1.69%)
Adrenal gland	3	(0.73%)

^a^57 patients without macrovascular invasion/extrahepatic metastasis but with intrahepatic metastasis after surgery were classified into CNLC III stage

HBV, hepatitis B virus

HCV, hepatitis C virus

AST, Aspartate transaminase

ALT, Alanine aminotransferase

AFP, alpha-fetoprotein

PVTT, portal vein tumor thrombus

HVTT, hepatic vein tumor thrombus

IVCTT, inferior vena cava tumor thrombus

In terms of tumor characteristics, the largest tumor diameter ranged from 0.5 to 20.5 cm. Tumor sizes ≤5 cm, >5–10 cm, and >10 cm accounted for 46.73%, 34.87%, and 18.40% of the cases, respectively. According to the China Liver Cancer Staging System (CNLC), 344 patients (83.29%) were classified as stage III. Macrovascular invasion (MVI) was identified in 270 patients (65.38%), most commonly involving the portal vein (149/270, 55.19%), followed by combined portal and hepatic vein involvement (104/270, 38.52%). Notably, extrahepatic metastasis occurred in 60 patients (14.53%), most commonly affecting the lungs (21/60, 35.00%).

### Efficacy

#### Tumor objective response to TILA-TACE

**Fig. 1:**
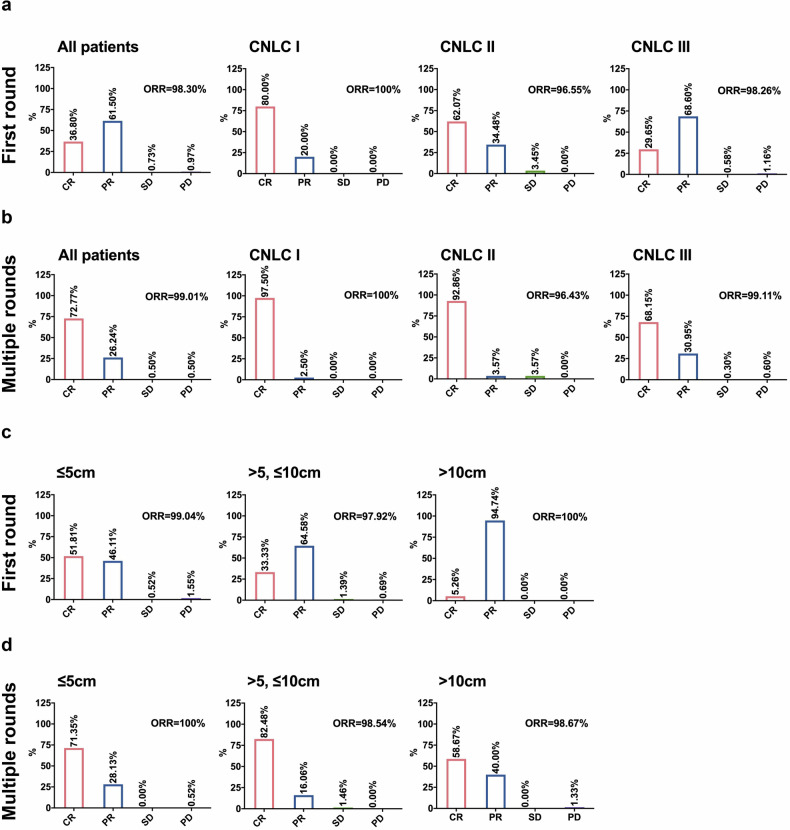
Tumor response rate to TILA-TACE. **a** Tumor response rate to a single round of TILA-TACE treatment. **b** Tumor response rate to multiple rounds of TILA-TACE treatment. **c** Response rate of tumors of different sizes to a single round of TILA-TACE treatment. **d** Response rate of tumors of different sizes to multiple rounds of TILA-TACE treatment

A total of 413 patients received TILA-TACE treatment. After the first round of treatment, the objective response rate (ORR) of the targeted tumors was 98.3%, including 36.8% complete response (CR), 61.5% partial response (PR), 0.7% stable disease (SD), and 1.0% progressive disease (PD) (Fig. 1a & Table S1). Among patients with early-stage HCC (CNLC I, equivalent to BCLC-A; n = 40), the ORR was 100% (80% CR, 20% PR). For intermediate-stage HCC (CNLC II, equivalent to BCLC-B; n = 29), the ORR was 96.6% (62.1% CR, 34.5% PR, 3.5% SD). In advanced-stage HCC (CNLC III, equivalent to BCLC-C; n = 344), the ORR was 98.3% (29.7% CR, 68.6% PR, 0.6% SD, 1.2% PD).

Following multiple rounds (Table S2) of TILA-TACE, the ORR increased to 99.01%, with 72.8% CR, 26.2% PR, 0.5% SD, and 0.5% PD (Fig. 1b & Table S3). In CNLC I patients, the ORR was 100%, with 97.5% achieving CR. Among CNLC II patients, the ORR was 96.4%, with 92.9% achieving CR. In CNLC III patients, the ORR was 99.1%, with 68.2% CR.

Conventional TACE is known to be less effective for large tumors.^26^ To evaluate whether the efficacy of TILA-TACE was similarly affected by tumor size, tumors were stratified into three groups: Group 1 ( ≤ 5 cm), Group 2 ( > 5 cm but ≤10 cm), and Group 3 ( > 10 cm). After the first round of treatment, the ORR was comparable across the three groups, but the CR rates (CRRs) varied markedly: 51.81% in Group 1, 33.33% in Group 2, and 5.26% in Group 3 (Fig. 1c). Multiple treatments substantially increased the CRR in all the groups, reaching values of 71.35%, 82.48%, and 58.67%, respectively (Fig. 1d). These results demonstrated that TILA-TACE was effective across tumors of varying sizes.

We found that the efficacy of TILA-TACE depended almost entirely on the vascular structure of the tumor and, technically, on the operability of its feeding arteries. Treatment outcomes can be categorized into four types:

1. Complete clearance in one treatment: When all tumor-feeding arteries were clearly defined by DSA and were technically suitable for TILA-TACE, complete tumor clearance was achieved in a single treatment.
2. Partial clearance during initial treatment: If all the tumor-feeding arteries were identified but some were initially inoperable (e.g., due to small inner diameters), partial clearance was achieved. The residual tumor tissues were cleared during subsequent treatments after the arteries enlarged sufficiently to accommodate the catheters.
3. Missed arteries identified in subsequent treatments: When some feeding arteries were not visualized during the initial DSA, only part of the tumor was treated. In subsequent DSA, the missed arteries were identified, and residual tumors were cleared in additional TILA-TACE sessions.
4. Partial clearance of large tumors: In very large tumors ( > 10 cm), TILA-TACE effectively cleared most of the tumor mass, but residual tissue that was not amenable to TILA-TACE remained. This limitation partly explains the lower CRR in larger tumors.

Taken together, these findings showed that the therapeutic efficacy of TILA-TACE was determined primarily by the accessibility and operability of tumor-feeding arteries. These four categories also highlighted the high selectivity of TILA-TACE, as evidenced by the sharply demarcated borders between the treated and untreated regions. Importantly, the efficacy and selectivity of TILA-TACE were observed across early, intermediate, and advanced stages of HCC.

#### Response of tumor vascular invasion and extrahepatic metastasis to radiation therapy

Among 270 patients with macrovascular invasion, 132 had identifiable tumor thrombus in the portal vein (PVTT), hepatic vein (HVTT), and/or inferior vena cava (IVCTT), whereas the remaining 138 had vascular invasion without detectable thrombus. Among the 132 patients with tumor thrombus, 131 received radiation therapy—primarily stereotactic body radiation therapy (SBRT)—and 128 underwent posttreatment imaging evaluation. The objective response rate (ORR) of tumor thrombus to radiation therapy was 96.88% (Table S4).

Among the subgroup of 138 patients with vascular invasion but no visible thrombus, 135 received radiation therapy, and 133 underwent MRI-based evaluations. Among these, vascular invasion was effectively controlled in 130 patients, with only 3 patients progressing to PVTT.

Among the 60 patients with extrahepatic metastasis, 35 received treatment including SBRT, or radioactive seed implantation. The treatment outcomes were as follows: 13 complete responses (CRs), 10 partial responses (PRs), 1 stable disease (SD), and 9 progressive diseases (PDs). Imaging data were unavailable for 2 patients and were therefore excluded from response evaluation (Table S5).

#### Overall survival

As of August 31, 2022, the median follow-up duration was 38 months overall. Specifically, the median follow-up was 36 months for CNLC I patients, 42 months for CNLC II patients, and 38 months for CNLC III patients.

**Fig. 2:**
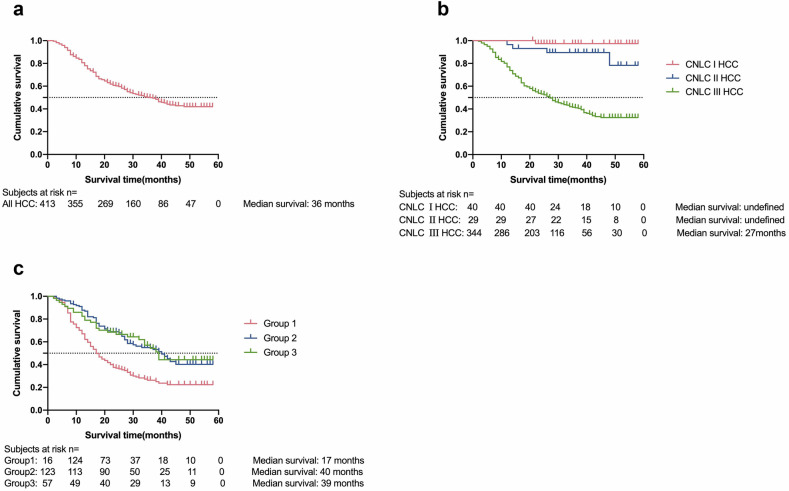
Kaplan‒Meier analysis of the cumulative survival of patients. **a** All the patients. **b** Survival of patients with different CNLC stages. **c** CNLC III patients were classified into 3 groups: group 1, with vascular tumor thrombus and/or extrahepatic metastasis; group 2, with vascular invasion but without observable vascular tumor thrombus; and group 3, with intrahepatic metastasis after the primary tumors were resected

During the follow-up period, the median overall survival (OS) was 36 months for 413 patients (Fig. 2a). One of 40 CNLC I patients, 4 of 29 CNLC II patients, and 204 of 344 CNLC III patients died. The estimated overall survival (OS) rates at 1, 2, and 3 years were as follows (Fig. 2b): CNLC I: 100%, 97.4%, and 97.4%; CNLC II: 96.6%, 93.1%, and 89.5%; and CNLC III: 77.0%, 53.1%, and 41.1%, respectively.

CNLC III HCC patients were further classified into three subgroups: subgroup 1, patients with vascular tumor thrombus and/or extrahepatic metastasis; subgroup 2, patients with vascular invasion but without observable vascular tumor thrombus; and subgroup 3, patients with intrahepatic metastasis following resection of the primary tumor.

The estimated overall survival (OS) rates at 1, 2, and 3 years were as follows (Fig. 2c): Subgroup 1: 67.0%, 36.9%, and 26.3%, with a median survival of 17 months; Subgroup 2: 87.8%, 68.9%, and 55.0%, with a median survival of 40 months; and Subgroup 3: 82.5%, 66.5%, and 54.3%, with a median survival of 39 months.

### Analysis of causes of death

We analyzed the causes of all 209 recorded deaths (Table 2). Among them, 152 (72.7%) were related to tumor progression, 51 (24.4%) were unrelated to tumor progression, 6 (2.9%) were of unknown cause, and 4 patients were lost to follow-up.

**Table 2:** Cancer progression-related death and nontumor progression-related death

	Patients	
**All**	**413**	
**Survival**	**200**	**(48.43%)**
**Death**	**209**	**(50.61%)**
*Tumor progression related death*	152	(36.80%)
Intrahepatic metastasis related	107	(25.91%)
Extrahepatic metastasis related	13	(3.15%)
Tumor thrombus related	3	(0.73%)
Gastrointestinal bleeding related	9	(2.18%)
Invasion of bile duct related	7	(1.69%)
Liver failure	10	(2.42%)
Not cooperative	3	(0.73%)
*Nontumor progression related death*	51	(12.35%)
*Unknown death*	6	(1.45%)
**Loss to follow-up**	**4**	**(0.97%)**

Among the 152 tumor-related deaths, 107 (70.4%) were attributed to intrahepatic metastasis. As illustrated in Fig. S1, despite complete responses of macrovascular invasion and primary tumors to SBRT and TILA-TACE, respectively, widespread intrahepatic metastases subsequently developed and progressed, even with systemic therapy.

**Fig. 3:**
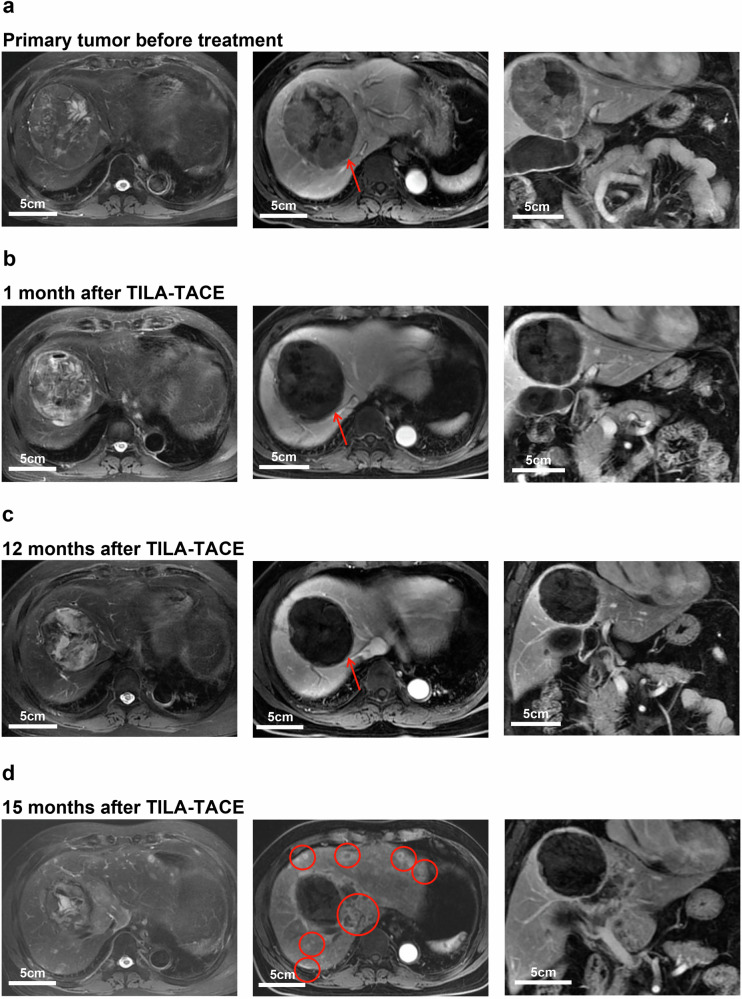
Representative MR images of subsequent intrahepatic metastasis of HCC after complete control of the targeted tumor (left: axial T2-weighted image (T2WI); middle: axial contrast-enhanced MRI; right: coronal MRI). **a** Scans before receiving TILA-TACE treatment, demonstrating T2 hyperintensity of the lesion (red arrow) and hyperenhancement on contrast-enhanced MR image compared with the background liver. **b** Scans one month after TILA-TACE treatment, demonstrating a complete necrotic lesion (red arrow). **c** Scans 12 months after TILA-TACE treatment, showing no new lesions or recurrences. **d** Scans 15 months after TILA-TACE treatment, showing that multiple intrahepatic metastases appeared (red circles)

These findings indicate that once local tumors and vascular invasion are effectively controlled, intrahepatic metastasis becomes the predominant cause of disease progression and death. Figure 3 presents a representative case of uncontrolled intrahepatic metastasis following complete response of the targeted tumor.

### Safety

A total of 413 patients received at least one round of TILA-TACE, with a median of 2 treatments (range: 1–9) (Table S2). Adverse events were reported in 375 patients (90.8%), primarily related to postembolization syndrome—including vomiting, abdominal pain, fever—as well as transient elevations in liver enzymes and bilirubin levels (Table 3 & Table S6). These adverse events were consistent with the known safety profile of conventional TACE.^6^ Metabolic alkalosis was not observed in any patient. All events were effectively managed with timely symptomatic treatment (Supplementary Methods).

**Table 3:** Adverse events

	Total	CTCAE≥grade 3			Possible causes
		Grade 3	Grade 4	Grade 5	
Fever	166	4	—	—	Postembolization syndrome
Abdominal pain	89	—	—	—	Postembolization syndrome
Nausea	19	—	—	—	Postembolization syndrome
Vomit	19	—	—	—	Postembolization syndrome
Bone marrow chemotherapy-related toxicity	3	2	—	—	TACE chemotherapy drug
Elevated creatinine	6	—	—	—	TACE radiocontrast agent
Skin ulcer/rash/erythema	3	—	—	—	TACE radiocontrast agent
Liver enzyme abnormalities	357	157	33	—	Postembolization syndrome
Bilirubin-related abnormalities	234	3	—	—	Postembolization syndrome
Liver abscess	2	2	—	—	TACE
Liquefactive necrosis of liver focus	2	2	—	—	TACE
Cholecystitis	1	1	—	—	TACE chemotherapy drug

The adverse events monitored also include liver decompensation (ascites, encephalopathy, and varices), hepatic arterial complications, hepatic failure, biliary disease, pleural effusion, gastrointestinal bleeding, duodenal ulcer, respiratory failure or decompensation, pulmonary embolism, seed migration, radioactive damage, and procedural complications. These events were not observed in the patients

With respect to liver function, the number of patients with Child‒Pugh grade A disease decreased from 365 to 336 after the first TILA-TACE procedure. However, following prompt supportive care, the number of Grade A patients increased to 389 after the first round and remained at 380 after multiple rounds (Table S6).

Together, these findings indicate that the side effects of TILA-TACE are manageable and acceptable.

### Subgroup analysis of the complete response rate (CRR)

Subgroup analyses were conducted to identify key predictors of the complete response rate (CRR) following initial and multiple rounds of TILA-TACE therapy (Table S7 & Table S8). After the first round of TILA-TACE, significant differences in the CRR were observed according to alpha-fetoprotein (AFP) level, Eastern Cooperative Oncology Group (ECOG) performance status, tumor size, and CNLC stage. Patients with an Eastern Cooperative Oncology Group (ECOG) score of 0 had a significantly greater CRR than those with an Eastern Cooperative Oncology Group (ECOG) score of 1 (72.5% vs. 29.7%; OR = 6.244, 95% CI: 3.507–11.114; p < 0.001). Tumor size was also a strong predictor: lesions ≤5 cm achieved a CRR of 51.8% (OR = 19.355 vs. >10 cm; p < 0.001). CNLC stage I patients exhibited a CRR of 80.0%, which was significantly greater than that of stage III patients (OR = 9.490; p < 0.001). In contrast, Child‒Pugh class (A vs. B: OR = 1.656; p = 0.141) and etiology (HBV vs. non-B/non-C: OR = 0.965; p = 0.900) were not significantly associated with the CRR.

Following multiple rounds of TILA-TACE, ECOG 0 status remained a strong predictor of a higher CRR (95.6% vs. 68.2%; OR = 10.124; p < 0.001), and CNLC stage I continued to be associated with superior outcomes (CRR = 97.5%; OR = 18.223 vs. stage III; p = 0.004). Notably, Child‒Pugh class A patients showed borderline significant improvement over class B patients (74.4% vs. 60.0%; OR = 1.935; p = 0.044), suggesting that preserved liver function may confer cumulative benefits with repeated treatment. AFP levels and etiology remained nonsignificant predictors of CRR after multiple rounds of treatment.

Importantly, patients who experienced recurrence or metastasis prior to receiving TILA-TACE had a significantly lower CRR following multiple rounds than did newly diagnosed patients (61.4% vs. 82.3%; OR = 2.916; p < 0.001).

## Discussion

In this study, 413 patients with hepatocellular carcinoma (HCC) were enrolled, including 40 with early-stage disease (9.7%), 29 with intermediate-stage disease (7.0%), and 344 with advanced-stage disease (83.3%). The findings provide two key insights. First, they reproduced the efficacy of TILA-TACE previously reported by our group,^25^ confirming the reproducibility of this treatment approach. Second, TILA-TACE demonstrated high efficacy across a heterogeneous tumor population encompassing various HCC stages and sizes, suggesting that this method could overcome therapy resistance commonly associated with tumor heterogeneity, which often limits the effectiveness of conventional TACE (cTACE).

To understand why TILA-TACE achieved such high response rates in a heterogeneous tumor population, we systematically analyzed the characteristics of all target tumors. Our analysis revealed that the efficacy of TILA-TACE was almost entirely determined by the vascular architecture of the tumors. Specifically, successful treatment depends on the technical feasibility of accessing and embolizing tumor-feeding arteries, which leads to four distinct treatment outcomes, as described in the Results section. This vascular dependency not only explains the consistently high efficacy of TILA-TACE across different tumor types but also accounts for its exceptional tumor-level response rate of 99.01%, consisting of 72.77% complete responses and 26.24% partial responses. The high rate of complete response was particularly noteworthy, as partial response often provides only transient tumor control, with a high likelihood of subsequent regrowth.

Owing to the lack of access to raw data from published studies, we used our own historical data as an external control and focused on the key differences between TILA-TACE and conventional TACE (cTACE), drawing on over 30 years of clinical experience with TACE at our institution. Two major distinctions emerged: efficacy and selectivity.

TILA-TACE frequently results in complete tumor necrosis, characterized by a sharp demarcation between the treated and untreated regions. When all the tumor-feeding arteries are successfully identified and embolized, the targeted tumor can be fully eradicated, yielding a clean boundary between the necrotic tumor tissue and the surrounding normal parenchyma. This mechanism underlies the high complete response rate (CRR) of 72.77% observed with TILA-TACE.

In contrast, cTACE typically produces a heterogeneous mix of necrotic and viable tumor tissues, often without a clear margin between treated and untreated areas. Even when all feeding arteries are technically accessible, complete tumor necrosis is rarely achieved with cTACE. Moreover, cTACE frequently results in indistinct boundaries between residual viable tumors and adjacent normal tissue, suggesting ongoing tumor infiltration into the surrounding parenchyma.^24,25,27^

Advanced-stage HCC accounts for approximately 30–35% of cases at initial diagnosis,^28–30^ and the median survival for these patients is typically less than six months.^31^ Improving survival in this population has been a long-standing challenge in hepatocellular carcinoma management. Sorafenib was the first systemic therapy shown to provide a statistically significant, although modest, survival benefit in advanced HCC, extending median survival by approximately 2–3 months compared with placebo.^32,33^ However, its objective tumor response rate is extremely low. In the pivotal phase III trial, only 2% of patients in the sorafenib group achieved a partial response, and none achieved a complete response; similar results were observed in the placebo group.^32^

Given the well-recognized limitations in the efficacy of sorafenib, there is a critical need for alternative treatment strategies to improve outcomes in patients with advanced HCC. Notably, Yoon et al. reported that, compared with sorafenib monotherapy, combining transarterial chemoembolization (TACE) with radiation therapy as a first-line treatment significantly improved progression-free survival, the objective response rate, the time to progression, and overall survival in patients with advanced HCC.^34^

In advanced HCC, we believe that a significant extension of patient survival can be achieved only when the tumors are effectively controlled. Intrahepatic tumors in advanced HCC generally consist of three types of lesions: primary tumors, tumor thrombus or vascular invasion, and disseminated lesions. These lesion types differ in their responsiveness to various treatment modalities. Specifically, primary tumors and well-vascularized disseminated lesions respond well to TILA-TACE, whereas tumor thrombus and poorly vascularized disseminated lesions respond poorly to TACE but are highly sensitive to stereotactic body radiotherapy (SBRT) or ^125^I seed implantation. Our results demonstrate that TILA-TACE effectively controls the primary tumor and well-vascularized disseminated lesions, whereas RT effectively controls the tumor thrombus, vascular invasion, and poorly vascularized lesions. The observed clinical efficacy represented the outcome of a targeted multimodal strategy in which each lesion subtype was treated with the most appropriate modality. Together, the comprehensive control of all three lesion types forms the foundation for the extended median survival of 27 months observed in patients with advanced HCC.

The effective control of all three lesion types also has critical therapeutic implications: it enables the identification of subsequent intrahepatic metastasis as the predominant cause of HCC-related death. This observation underscores the urgent need to develop effective strategies specifically targeting intrahepatic dissemination to further improve the overall survival of patients with HCC.

In this study, 40 patients with CNLC stage I HCC were treated with TILA-TACE, achieving a complete response rate of 97.5% and a partial response rate of 2.5%, with only one death reported during a median follow-up period of 38 months. Similarly, previous studies have demonstrated that TACE could serve as an effective alternative treatment for BCLC stage 0/A HCC patients who are not candidates for curative therapies, providing long-term survival benefits.^35–38^ These findings support the notion that TACE, particularly TILA-TACE, might be a viable therapeutic option for patients with early-stage HCC who are unwilling or unable to undergo surgery because of factors such as advanced age, limited hepatic functional reserve, tumors located in high-risk anatomical regions, impaired cardiopulmonary function, severe cirrhosis, or other comorbidities.

In this study, 225 patients were newly diagnosed with HCC, whereas 188 had recurrent disease. A subgroup analysis was conducted to evaluate the impact of prior treatment history on overall survival, revealing no significant difference between the two groups (Fig. S2). The recurrent patients had previously undergone treatments such as surgical resection, ablation, conventional transarterial chemoembolization (cTACE), or drug-eluting bead TACE (DEB-TACE), all of which were administered at other institutions. These patients were included in the present study because they either developed new intrahepatic lesions or experienced recurrence of previously treated tumors. Notably, the target lesions treated with TILA-TACE in this study were either treatment naïve or resistant to prior local or regional therapies. This finding suggested that the therapeutic efficacy of TILA-TACE was independent of previous treatment history.

This study confirms the efficacy and safety of TILA-TACE previously reported by our group.^25^ However, a limitation is that the findings are based on a single-center experience. To establish broader clinical relevance, multicenter trials are needed to further validate the utility of TILA-TACE as a treatment option for HCC, particularly advanced-stage disease. In parallel, other combination strategies, such as lenvatinib plus TACE (LEN-TACE), have also demonstrated encouraging results in prospective multicenter trials, including TACTICS-L and LAUNCH.^39,40^ These emerging therapies, including TILA-TACE and LEN-TACE, may offer complementary strategies for improving outcomes in patients with HCC and warrant continued comparative and combinatorial investigations.

## Methods

### Study design

#### Patients

This study was conducted with written informed consent obtained from all patients and approval from the hospital’s Institutional Review Board (ChiCTR registration number: ChiCTR-ONC-17013416). A total of 413 patients were enrolled between November 2017 and December 2020 (Table 1 & Fig. S3). Hepatocellular carcinoma (HCC) staging was performed according to the Guidelines for Diagnosis and Treatment of Primary Liver Cancer in China.^4,5^ Patients were categorized as early-stage HCC (CNLC I, equivalent to BCLC A), intermediate-stage HCC (CNLC II, equivalent to BCLC B), and advanced-stage HCC (CNLC III, equivalent to BCLC C), representing 9.7%, 7.0%, and 83.3% of the cohort, respectively. Among them, 225 patients were newly diagnosed, while 188 had recurrent disease.

Advanced HCC patients were further classified into three groups: Group 1 included those with vascular tumor thrombus and/or extrahepatic metastasis; Group 2 included patients with vascular invasion but without observable tumor thrombus; and Group 3 consisted of patients with intrahepatic metastasis without observable vascular tumor thrombus or extrahepatic metastasis. Detailed demographic and clinical characteristics are summarized in Table 1.

Inclusion criteria comprised: diagnosis of HCC based on EASL guidelines and/or histological confirmation,^41^ CNLC stage I–III, Child‒Pugh class A or B, and presence of hypervascular lesions as assessed by triphasic MRI. Exclusion criteria included CNLC stage IV, Child‒Pugh class C, or evidence of combined arterio-venous shunt.

#### Treatment rationale

##### CNLC I HCC

TILA-TACE was applied to treat patients with CNLC I stage HCC in accordance with the Guidelines for the Diagnosis and Treatment of Hepatocellular Carcinoma in China.^4,5^ While TACE is recommended for early-stage CNLC-1b patients, it may also be considered for CNLC-1a patients who are eligible for surgical resection or ablation but decline or are unable to undergo these treatments owing to nonsurgical factors. These factors include advanced age, limited liver function reserve, tumors located in high-risk areas, impaired cardiac or pulmonary function, severe cirrhosis, or other comorbidities.

##### CNLC II HCC

TILA-TACE was also used to treat CNLC II HCC, as TACE is the recommended treatment for this stage according to the Chinese guidelines.^4,5^

Given the high efficacy of TILA-TACE for local tumor control demonstrated in our previous studies,^25^ this treatment was applied to patients with CNLC I and II HCC.

##### CNLC III HCC

CNLC III HCC encompasses three types of intrahepatic lesions: the primary tumor, tumor thrombus or vascular invasion, and disseminated lesions. These lesion types respond differently to various treatment modalities. On the basis of our prior experience, primary lesions and well-vascularized disseminated lesions respond favorably to TILA-TACE, whereas tumor thrombus and poorly vascularized disseminated lesions respond poorly to TACE but are highly sensitive to stereotactic body radiotherapy (SBRT) or ^125^I seed implantation. This finding provides a rationale for combining TILA-TACE with radiotherapy to treat advanced-stage HCC. The combination of TACE and radiation therapy is also recommended by the Chinese guidelines for CNLC III stage HCC.^4,5^

For CNLC III patients, several considerations were made to evaluate the respective efficacies of TILA-TACE and radiation therapy. First, TILA-TACE was employed to control the primary tumors, whereas SBRT targeted tumor thrombus in the main, left, and right portal veins as well as the inferior vena cava. Radioactive ^125^I seeds have been used to treat tumor thrombus in liver segments (e.g., VP2) and intrahepatic disseminated lesions. The distinct anatomical locations of these lesions enabled precise, modality-specific targeting by TILA-TACE, SBRT, and radioactive seeds. Second, there was a time interval between TILA-TACE and SBRT. Third, the necrosis pattern induced by TILA-TACE, as observed via MRI, is characterized by a clear and well-defined border separating treated areas from untreated areas,^24,25,27^ a feature readily recognized by radiologists and TACE specialists.

##### TILA-TACE

The detailed criteria for TILA-TACE treatment are described in the supplementary methods. The TILA-TACE procedure was described in detail in our previous report.^25^ Briefly, TILA-TACE was performed through the transfemoral route via a 5-Fr catheter (Shepherd-hook modified angiographic catheter, HANACO Medical, Tian Jin, China) that was advanced to the celiac artery. The tumor-feeding arteries were identified via digital subtraction angiography (DSA) via a coaxial microcatheter. A coaxial microcatheter (1.98Fr, ASAHI Masters PARKWAY SOFT, ASAHI INTECC, Japan; 2.4Fr, MAETRO, Merit Medical, USA) was selectively inserted into the tumor feeding artery, into which, 5% sodium bicarbonate (5% Sodium Bicarbonate Injection, Hunan Kelun Pharmaceutical, Ltd., Hunan, China) was infused alternatively with doxorubicin-lipiodol emulsion and oxaliplatin/homocamptothecin with the dose adjusted to tumor size. Finally, the tumor-feeding artery was embolized with microspheres of proper size (Embosphere®, BioSphere Medical, Paris, France) and/or a microcoil (Tornado®, COOK Medical, USA).

During arterial infusion of sodium bicarbonate into tumor-feeding arteries, some patients may experience transient distending pain in the liver region. This pain depends on whether the tumor-feeding artery supplies only the tumor or branches to normal liver tissue. If the artery exclusively supplies the tumor, pain is typically absent. However, if it also perfuses normal tissue, the infusion may cause discomfort. This pain usually resolves immediately upon stopping the infusion. To improve patient comfort, we routinely administer 2–3 mL of a diluted lidocaine solution (prepared by mixing 0.1 g/5 mL lidocaine with 15 mL of normal saline) via arterial injection prior to infusion. This pretreatment effectively prevents or alleviates pain. If pain persists during the infusion, an additional 2–3 mL dose of the diluted lidocaine solution can be administered for further relief.

##### SBRT

SBRT is used for the treatment of tumor thrombus in the main, left and right portal veins and the inferior vena cava. A stereotactic body frame (Karity, Guangzhou, China) was used to immobilize the patients with the assistance of vacuum bags. For each patient, planning four-dimensional contrast-enhanced computed tomography (4DCT) was performed. The gross tumor volume (GTV) was strictly defined according to the morphology of the tumor thrombosis visualized on contrast-enhanced CT and MRI. The internal target volume (ITV) was determined as the combined volume of GTVs in the multiple 4DCT phases. The planning target volume (PTV) was added with 3–5 mm margins from the ITV. The mean volume of the PTV was 166.0 ± 143.8 cm^3^ (20.8–849.0 cm^3^). The median prescription dose to the PTV was 45 Gy (range, 35–56) in five fractions.

##### Radioactive seed implants

The radioactivity of ^125^I seeds was 27.4–35.5 keV/particle, with a half-life of 60.1 days (Xinke, Shanghai, P. R. China). The radioactive seeds are used for treating tumor thrombus in vessels located in segments of the liver, such as VP2, as well as intrahepatic disseminated tumors. The length of each seed was 4.5 ± 0.2 mm, with an effective penetration range of 17 mm. The activity of the ^125^I seeds used for implantation was 0.65 millicuries (mCi) per seed. The total dose of radioactivity was the radiation dose of a single particle multiplied by the number of implanted particles. It was stored in a titanium container at room temperature. Under CT guidance, an 18-gauge implant needle was placed in the target lesion. A seed implantation gun (Xinke, Shanghai, P. R. China) was used to implant ^125^I seeds with a 0.5–1.0 cm distance between two seeds.

##### Other therapies

Systemic therapy (targeted therapy and immunotherapy) was only used for intrahepatic metastases (for those patients whose metastatic tumor lesions ≥ 5) that occurred after the primary tumors and tumor vascular invasion were controlled by TILA-TACE and radiation therapy. Patients received oral lenvatinib 12 mg/day (for body weight ≥60 kg) or 8 mg/day (for body weight <60 kg), sorafenib 400 mg/day, and 200 mg tirelizumab every 3 weeks (intravenous injection). Dose reductions for lenvatinib/sorafenib-related toxicities were permitted. The drugs should be discontinued in cases of severe side effects or disease progression.

### Efficacy assessment

The assessment was performed independently by 3 radiologists.

#### Tumor response to TILA-TACE

The response of the target tumor to treatment was assessed 30 days after the first treatment and at every follow-up visit via MRI, which was specifically designed to assess the viable portion of the tumor, as defined by arterial phase enhancement on dynamic imaging, on the basis of mRECIST criteria.^42^ The tumor response to treatment was graded as follows: complete response (CR), complete necrosis; partial response (PR), ≥30% decrease in viable residues; stable disease (SD), any cases that do not qualify for either partial response or progressive disease; and progressive disease (PD), ≥20% increase in viable tumors.

#### MVI response to treatment

The diagnosis of MVI^43,44^ was based on the demonstration of vascular invasion on MRI (including invasion of the portal vein, hepatic vein or inferior vena cava). MVI with a tumor thrombus was defined as enhancement of the thrombus in the arterial phase, filling defects in the venous phase, and high signal intensity in diffusion-weighted images and T2-weighted images. When imaging features do not establish the presence of a tumor thrombus, there are several additional clues for the diagnosis of MVI, including occluded veins with ill-defined walls or restricted diffusion, occluded or obscured veins contiguous with malignant parenchymal masses, and heterogeneous vein enhancement not attributable to artifacts according to the Liver Imaging Reporting and Data System (LiRADs v2018). The response of the PVTT, HVTT, and IVCTT to treatment was evaluated at the final follow-up visit. The efficacy response was assessed by the signal intensity change in diffusion-weighted images and T2-weighted images, which was mainly based on the mRECIST criteria. The response of MVI without observable tumor thrombus to treatment was also scored by MRI.

#### Extrahepatic metastasis response to treatment

Efficacy was assessed after treatment on the basis of the mRECIST criteria.

#### Overall survival (OS)

OS was measured from the date of the first treatment until the date of death from any cause or the final follow-up visit. The median follow-up was 38 months, ranging from 2–58 months (calculated from the date of the first treatment until the date of death caused by any reason or the final follow-up visit).

### Endpoints

In our previous study,^25^ the local efficacy of TILA-TACE was well established, with an objective response rate (ORR) of 100%. Building on these findings, the current real-world study aimed to evaluate the long-term clinical benefit of this technique. Therefore, we selected overall survival (OS) as the primary endpoint, as it best reflects the true therapeutic value and durability of the treatment strategy. The secondary endpoint was the tumor response rate.

### Adverse events (AEs)

The adverse events monitored included fever, abdominal pain, nausea and vomiting, skin ulcers, liver enzyme abnormalities, bilirubin-related abnormalities, liver abscess, liquefactive necrosis of the liver focus, liver decompensation (ascites, encephalopathy, and varices), hepatic arterial complications, hepatic failure, biliary disease, pleural effusion, gastrointestinal bleeding, duodenal ulcers, respiratory failure or decompensation, pulmonary embolism, bone marrow chemotherapy-related toxicity, renal dysfunction, seed migration, radioactive damage, and procedural complications. The adverse events were scored according to the Common Terminology Criteria for Adverse Events version 5.0 in the supplementary methods. All adverse events received prompt symptomatic treatment.

### Statistics

SPSS version 25.0 statistical software and GraphPad Prism version 8 were used for all the statistical analyses. Survival curves for time‒to-event variables were generated via the Kaplan‒Meier method, and comparisons of survival curves were performed via the log-rank test. The CRR, ORR, and 95% CI were calculated via the Clopper‒Pearson method. We conducted prespecified subgroup analyses of previous history (first diagnosed, recurrent or metastatic), viral status (hepatitis B and/or hepatitis C seropositivity versus nonviral etiology), alpha-fetoprotein level ( ≤ 20 ng/mL, >20–400 ng/mL, and >400 ng/mL), Child‒Pugh class (A or B), Eastern Cooperative Oncology Group (ECOG) score (0 or 1), size of the largest tumor ( ≤ 5 cm, >5–10 cm, and >10 cm), and CNLC stage to assess the consistency of the CRR across various subgroups. We used logistic regression to compare the difference in the CRR between each subgroup. For all tests, a p value less than 0.05 was considered statistically significant.

## Supplementary information

TILA-TACE study protocol

Supplementary file

## Data Availability

All data should be available once this manuscript released.
