# Supplementary material for: Bicarbonate-integrated transarterial chemoembolization (TACE) in real-world hepatocellular carcinoma
Source: Signal Transduct Target Ther. 2025 Sep 1;10:281. doi: 10.1038/s41392-025-02400-x (PMC12399753; doi:10.1038/s41392-025-02400-x)
Supplement: Supplementary file 1 — TILA-TACE study protocol [file 41392_2025_2400_MOESM1_ESM.pdf]

TILA-TACE 治疗肝细胞肝癌的前瞻性单臂研究

方案编号：**ZUSAHZUCI201701**

版 本 号：**V3.0**

版本日期：**2017** 年 **11** 月

临床试验负责单位：浙江大学附属第二医院

参加单位及签字页

我已阅读了此方案，方案编号 **ZUSAHZUCI201701**，版本号 V3.0（版本日期：2017 年 11 月），我同意此方案的内容，并同意按此方案进行临床研究。

我将对此方案及相关内容保密。

申请人：

负责人（签字）：　　　　　　　签字日期：　　年　　月　　日

临床研究组长单位：　浙江大学附属第二医院

主要研究者（签字）：　　　　　签字日期：　　年　　月　　日

统计单位:　浙江大学肿瘤研究所

负责人（签字）：　　　　　　　签字日期：　　年　　月　　日

目　　录

方案摘要 3
缩略语表 5
1 研究背景 6
2 研究目的和主要观察指标 7
3 研究设计 8
3.1 总体设计 8
3.2 研究人群 8
3.2.1 受试者及研究例数 8
3.2.3 排除标准 8
3.3 试验设计 9
3.4 TILA-TACE 术中给药方案 10
3.5 退出试验的标准 10
3.5.1 研究者决定的退出 10
3.5.2 受试者自行退出试验 10
3.5.3 终止试验的标准 11
3.5.4 剔除标准 11
4 试验具体步骤 11
5 试验结束后随访评估 13
6 安全性评价 13
7 伦理规范及知情同意 13
8 伦理规范 13
8 知情同意 13
8 应急措施 13
9 临床试验的质量保证 13
10 数据处理及资料保存 14
10.1 病例报告表（CRF） 14
10.2 统计分析 14
11 不良事件呈报 14
12 临床试验进度 15

方案摘要

研究题目：	**TILA-TACE 治疗肝细胞肝癌的前瞻性单臂研究**
试验目的：	验证 TILA-TACE 的临床疗效
试验方法：	单臂临床研究
首要观察指标：	生存率,中位生存时间
次要观察指标：	肿瘤对治疗的反应率
入组标准：	1.经病理组织证实或符合相应临床诊断标准，明确诊断的原发性肝细胞癌患者；<br>2.体力状况 ECOG 评分 0-1 分；<br>3.肝癌 BCLC 分期：0, A, B 期或 C 期；<br>4.术前 Child-Pugh 评分：A 级或 B 级；<br>5.经研究者判断，患者能遵守试验方案；<br>6.患者自愿参加本次临床试验，理解研究程序且能够书面签署知情同意书。
排除标准：	1. 肝癌 BCLC 分期： D 期；<br>2．术前 Child-Pugh 评分：C 级；
给药方案：	将符合入组标准并接受肝动脉插管化疗性栓塞的肝癌患者在接受常规肝动脉插管栓塞同时，经导管向瘤内注入 5% 碳酸氢钠注射液。
	**经肝动脉插管化疗栓塞/栓塞方案：**<br>常规消毒铺巾，经股动脉置入 5F 动脉鞘后，先采用 5F 牧羊钩造影导管（Shepherd-hook modified Angiographic Catheter, HANACO Medical, Tian Jin, China）勾选腹腔动脉造影。随后采用 2.8F 同轴微导管（Marguerite II, ASAHI INTECC GMA CO., LTD, Nagoya, Japan）分别超选择至肝总动脉、肝固有动脉、左右肝动脉以及肿瘤滋养动脉进行造影，明确肿瘤血供情况。在透视下，超选择至每支肿瘤滋养动脉，采用 5%碳酸氢钠注射液、吡柔比星-碘油混悬液及化疗药物交替使用进行化疗性栓塞（首先采用 5%碳酸氢钠注射液经导管注入肿瘤滋养动脉，随后采用吡柔比星-碘油混悬液经微导管注入肿瘤滋养动脉进行栓塞，同时配合奥沙利铂注射液以及羟基喜树碱注射

	液对肿瘤进行动脉灌注化疗，交替进行）。栓塞满意后，采用合适大小的PVA 颗粒（大小范围：100-900um，Embosphere®, BioSphere Medical, Paris, France）以及微弹簧圈（Tornado ®, COOK Medical, USA）对肿瘤滋养动脉进行彻底断流。栓塞完成后再次全面造影证实肝内无异常肿瘤染色后结束治疗。<br>**辅助治疗**<br>对有肝内和肝外转移，脉管侵犯的病人可能需要辅助治疗，包括但不局限于CRT，SBRT，SIRT<br>**随访：**<br>定期 MRI 检查，定期随访生存期。
**统计方法：**	所有统计分析将采用 Graphpad Prism 分析软件。。
**试验进度：**	预计试验开始时间：2017 年 11 月<br>预计试验结束时间：2023 年 12 月
**版本号：**	V1.0

缩略语表

TILA-TACE	靶向肿瘤内乳酸的经肝动脉插管化疗性栓塞
HCC	肝细胞肝癌
DSA	数字减影血管造影机
ECG	心电图

1 研究背景

首先，肝细胞肝癌是世界范围内第六大常见恶性肿瘤，并且居肿瘤致死率第 3 位 [1]。绝大多数肝细胞肝癌患者在发现时已属晚期，无法接受根治性治疗（外科手术切除或肝移植），因此，经肝动脉插管化疗栓塞术（TACE）是这些患者接受的主要治疗方式 [2-3]。但是 TACE 治疗后的完全肿瘤反应率仍然极低（0-4.8%）[4]。

为提高 TACE 的疗效，我们根据我们前期的发现的生物化学原理，将碳酸氢钠加入 TACE 治疗程序中。高乳酸环境是大多数实体肿瘤微环境的因子。我们发现高乳酸环境能保护代谢胁迫下的肿瘤细胞 [5-6]。而我们最近的研究表明，乳酸酸中毒是赋予肿瘤细胞抵抗葡萄糖剥夺的一个强有力因子：在乳酸酸中毒情况下，肿瘤细胞，例如 4T1 细胞，可以在葡萄糖剥夺的情况下存活 65 天，而在没有乳酸酸中毒的情况下，肿瘤细胞在 3 天内就会死亡。此外，在乳酸酸中毒情况下，肿瘤细胞表现为非 Warburg 表型，即对于葡萄糖的利用更为经济有效，即使是非常有限的葡萄糖，就可以维持肿瘤细胞的存活，反之亦然。值得指出的是，乳酸酸中毒对于肿瘤细胞的效应依赖于乳酸盐和氢质子的共存，去除两者中的任何一个将终止乳酸酸中毒对于肿瘤细胞的保护作用。当乳酸被碱性物质中和，例如碳酸氢钠，乳酸就变成了乳酸盐（高浓度的乳酸盐同时伴有碱性的 PH 值），乳酸就失去了上述效应，而酸中毒（低浓度的乳酸盐伴有酸性的 PH 值）仅能够提供肿瘤细胞对于葡萄糖剥夺非常有限的保护作用。

由于 TACE 通过阻断肿瘤滋养动脉减少肿瘤血供，同时将高浓度的化疗药物输送到肿瘤局部起到杀伤肿瘤细胞的作用。而高乳酸环境则能对抗 TACE 造成的代谢胁迫（营养剥夺）因此能降低 TACE 的疗效。反过来，碱如碳酸氢钠能中和乳酸，原理上能显著增加 TACE 疗效。

我们在先前一项队列研究中，采用靶向肿瘤内乳酸的经肝动脉插管化疗栓塞术（TILA-TACE）治疗了 40 例肝癌患者，取得了比常规 TACE（cTACE）更好的治疗效果，而两组患者之间不良事件的发生率并没有显著性差异。进而，我们做了随机对照研究，进一步说明了 bTACE 对肝细胞肝癌的疗效显著优于 cTACE[7]。

这两项临床研究说明了碳酸氢钠中和肿瘤 lactic acidosis 对肝细胞肝癌临床治疗的

重要作用。

本研究将用大样本验证 TILA-TACE 的临床疗效.

参考文献

1. Parkin DM, Bray F, Ferlay J, Pisani P. Global cancer statistics, 2002. CA Cancer J Clin. 2005; 55(2): 74-108.
2. Llovet JM, Bruix J. Systematic review of randomized trials for unresectable hepatocellular carcinoma: Chemoembolization improves survival. Hepatology. 2003; 37(2): 429-42.
3. Forner A, Llovet JM, Bruix J. Hepatocellular carcinoma. Lancet. 2012; 379(9822): 1245-55.
4. Jansen MC, van Hillegersberg R, Chamuleau RA, van Delden OM, Gouma DJ, van Gulik TM. Outcome of regional and local ablative therapies for hepatocellular carcinoma: a collective review. Eur J Surg Oncol. 2005; 31(4): 331-47.
5. Wu H, Ding Z, Hu D, Sun F, Dai C, Xie J, et al. Central role of lactic acidosis in cancer cell resistance to glucose deprivation-induced cell death. J Pathol. 2012; 227(2): 189-99.
6. Xie J, Wu H, Dai C, Pan Q, Ding Z, Hu D, et al. Beyond Warburg effect - dual metabolic nature of cancer cells. Sci Rep. 2014; 4: 4927.
7. Chao M, Wu H, Jin K, Li B, Wu J, Zhang G, Yang G3, Hu. A nonrandomized cohort and a randomized study of local control of large hepatocarcinoma by targeting intratumoral lactic acidosis. Elife. 2016: e15691

2 研究目的和主要观察指标

研究目的：

验证肿瘤反应率: 我们前期的研究得到 TILA-TACE 取得了 100%的肿瘤反应率。然而，前期研究的样本量只有 40 例，因此需要大样本来验证。

验证病人生存期：我们前期的研究指出，TILA-TACE 治疗了 40 例大肝癌的病人，这些病人的中位生存期大于 41 个月。前期研究的样本量只有 40 例，因此需要大样本来验证。

主要观察指标：

术后 30 天肝脏增强 MRI 上测得的肿瘤残留体积。根据残留肿瘤体积将治疗效果分成 5

个等级：完全反应（没有明确的肿瘤存活），近完全反应（肿瘤残活量小于 10%），部分反应（肿瘤残活量大于等于 10%，小于 50%），无反应或无进展（肿瘤残活量大于等于 50%，小于 100%），肿瘤进展 （肿瘤存活量大于等于 100%）。

记录病人的生存期。

3 研究设计

3.1 总体设计

本研究为验证 TILA-TACE 的临床有效性。为前瞻性的单臂研究。

3.2 研究人群

3.2.1 受试者及研究例数

本临床试验将纳入肝细胞肝癌患者作为研究对象。预计纳入 2000 例。

3.2.2 入选标准

1.经病理组织证实或符合相应临床诊断标准，明确诊断的原发性肝细胞癌患者；

2.体力状况 ECOG 评分 0-1 分；

3.肝癌 BCLC 分期：0, A, B 期或 C 期；

4.术前 Child-Pugh 评分：A 级或 B 级；

5.经研究者判断，患者能遵守试验方案；

6.患者自愿参加本次临床试验，理解研究程序且能够书面签署知情同意书。

3.2.3 排除标准

1. 肝癌 BCLC 分期： D 期；

2． 术前 Child-Pugh 评分 C 级；

3.3 试验设计

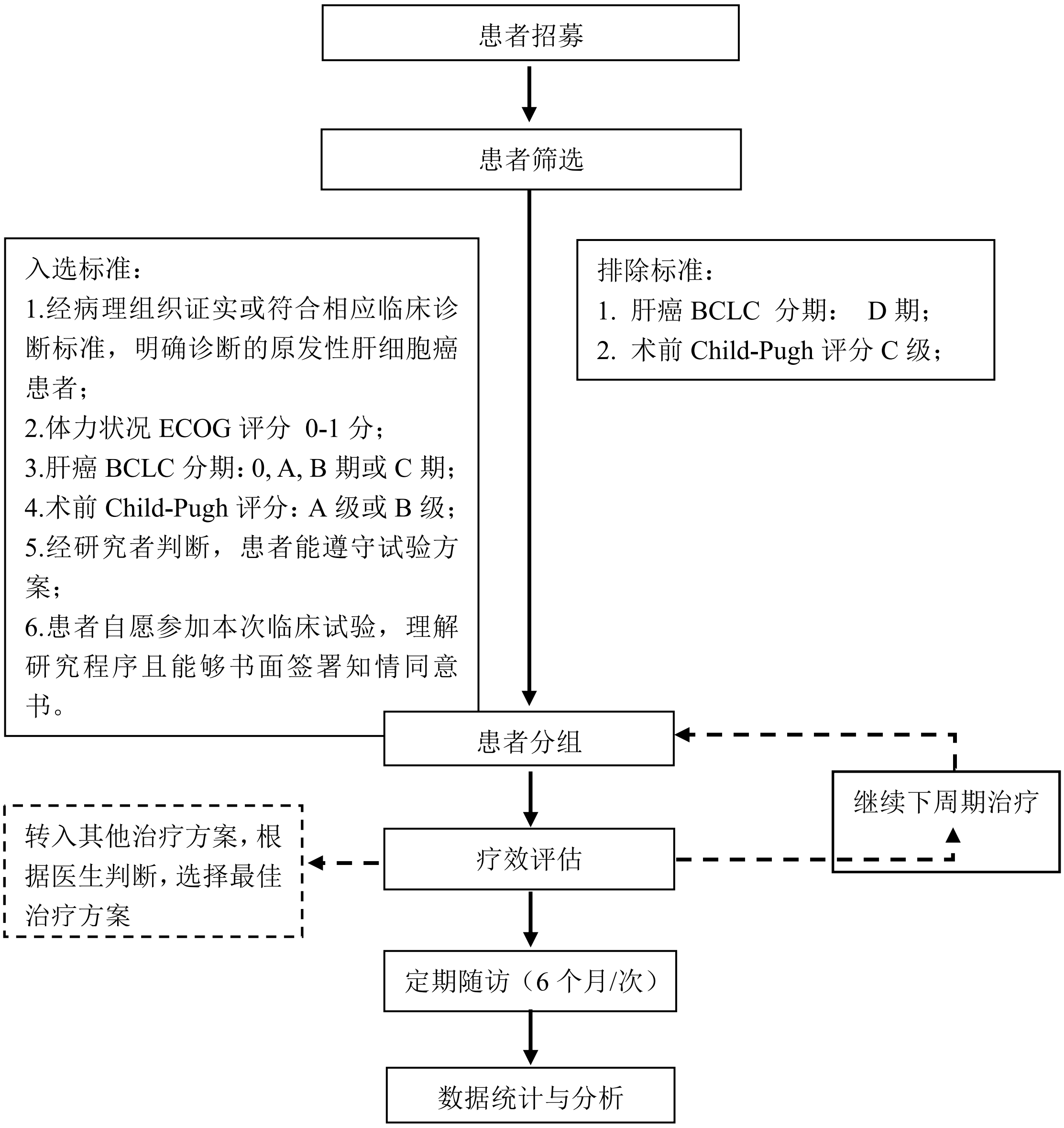

3.4 TILA-TACE 术中给药方案

盐酸吡柔比星注射液：奥沙利铂注射液注射液：羟基喜树碱注射液：超液化碘油；5%碳酸氢钠注射液。

注：1. 上述用药均为经肝动脉灌注化疗；

2. 化疗药物、超液化碘油、5%碳酸氢钠注射液实际用总量根据DSA造影结果以及患者肝功能情况由手术医师决定实际用量。

辅助治疗： 对有肝内外转移、癌拴的辅助治疗包括但不限于 3D-CRT, SBRT, SIRT。

3.5 退出试验的标准

3.5.1 研究者决定的退出

退出试验是指已经入选的受试者在研究过程中出现了不宜继续进行试验的情况下，研究者决定该病例退出试验。

1）发生严重不良事件，研究者判断不适宜继续接受试验的受试者；

2）受试者依从性差，影响试验结果判定者，如受试者不按规定进行检查者；

3）受试者试验过程中出现疾病复发或进展；

3.5.2 受试者自行退出试验

受试者不愿继续参加临床试验，根据知情同意书的规定，受试者有权中途退出试验，或受试者虽未明确退出试验，但不再接受用药及检测而失访（也属于退出，或称脱落）。应尽可能了解其退出的原因，并加以记录。如：对某些不良事件感到难以耐受；有事不能继续接受临床研究；经济因素；或未说明原因等。

3.5.3 终止试验的标准

终止试验是指临床试验尚未按方案结束，中途停止全部试验。若在试验终止前，已得到充分的数据可验证 TILA-TACE 的有效性，就可终止试验。终止的目的主要是为了保护受试者权益，保证试验质量，避免不必要的经济损失。提前终止临床试验应及时书面通知患者及研究各方。

3.5.4 剔除标准

资料统计分析前，由统计人员及主要研究者讨论判断个例是否剔除。出现下列情况之一，统计人员和主要研究者应依据受试者完成试验的程度和退组原因等因素综合判断是否将此受试者剔除，不再纳入最终统计，并作出相关说明。

（1）不符合入组条件者。

（2）未行规定的检查。

（3）疾病进展，无法耐受或不适合再次进行 TACE 术。

4 试验具体步骤

1) 伦理委员会审批临床试验方案、知情同意书等试验相关材料。

2) 试验相关材料通过伦理委员会审批后，开始进行受试者招募工作，收集对招募作出回馈的患者信息，包括但不局限于：人口统计学资料，病史，过敏史，用药史，献血史，之前参加临床试验情况；患者身份证、通讯地址、联系电话。

3) 筛选期步骤：

①召集基本信息符合试验要求的患者进行筛选，患者参加筛选前必须本着自愿的原则书面签署知情同意书。筛选工作在给药第-7 天至第-1 天进行，其中血清学检查（包括乙肝五项、HCV 抗体检测、HIV 抗体检测）和肝脏 MRI 结果在给药-30 至-1 天内有效，符合入选标准的患者方可参加试验。使用含表阿霉素的方案前可以根据心电图结果选择是否进行心脏超声检查。

②给药前筛选需要完成的检查包括：

- ECOG 评分
- 生命体征
- 体格检查
- 心电图
- 实验室检查：血、尿、便常规、肝肾功能、血清 AFP、凝血常规、大便隐血检查；乙肝五项、HCV 抗体检测、HIV 抗体检测。
- 影像学检查：胸部 CT 平扫、肝脏增强 MRI、肝脏 CT 平扫。

4) 每组受试者按照随机分组接受给药，试验结束后进行有效性和安全性评估。

5) 试验期间每个周期进行的检查包括：

- ECOG 评分
- 生命体征
- 体格检查
- 心电图
- 实验室检查：血、尿、便常规、肝肾功能、血清 AFP、凝血常规、大便隐血检查；血清学检查（包括乙肝五项、HCV 抗体检测、HIV 抗体检测）。
- 影像学检查：胸部 CT 平扫、肝脏增强 MRI、肝脏 CT 平扫。
- 不良事件记录：记录化疗期间发生的任何不良事件。
- 说明：原则上尽量使用本院的检查报告，特殊原因外院化验单可接受，但必须保存化验单原件。

5 试验结束后随访评估

本试验结束后，可以由研究者和受试者共同决定下一步治疗，并作好记录。

6 安全性评价

以下在 TACE 术中及术后可能出现的不良事件将会被监测：TACE 术中的血压、氧饱和度；TACE 术后疼痛、发热、肝功能变化；所有不良事件将会被记录。

7 伦理规范及知情同意

8 伦理规范

本试验方案实施前需经“浙江大学医学院附属第二医院伦理委员会”审核批准，方可开始本项研究。在临床研究期间，本试验方案做一定的修改均应向伦理委员会报告并备案。

8 知情同意

受试者在接受本试验治疗前必须对参加本试验知情同意，以保障受试者的合法权益。研究者有责任向受试者或其指定代表人完整、全面地介绍本研究的目的、药物的作用、可能出现的毒副反应和可能的风险，应让受试者知道他们的权利，所要承担的风险和受益。谈话是十分重要的知情同意过程。如受试者和其合法代表无识字能力，知情同意过程应有见证人参加，由受试者或其合法代表口头同意后，在知情同意书上签名，见证人的签名应与受试者的签名在同一天。知情同意书应注明版本和制订日期或修改日期。

8 应急措施

试验场所必须配备必要的医疗抢救设备、急救药品以及应急措施。

9 临床试验的质量保证

- 负责获得每一位受试者或其代理人签署的《知情同意书》。

- 认真按照要求填写病例报告表（CRF）。

- 完整保留实验室检查的记录、临床记录以及受试者的原始医疗记录。

10 数据处理及资料保存

10.1 病例报告表（CRF）

病例报告表（CRF）是临床试验中临床资料的记录方式。研究者对每位受试者在试验中的所有相关资料均应及时、真实地记录在案，并做好确认和签名。CRF 作为原始资料，不得随意更改，确实需要更改时研究者应签名并注明日期（见填写说明）。CRF 一式三联，试验结束后分别交给研究者、研究者所在医院的 GCP 中心和申办者保存。完成的 CRF 由临床监查员审查后，进行数据录入，内容不再修改。为保证受试者的隐私权，在 CRF 上的受试者姓名需使用代码。

10.2 统计分析

10.2.1 数据收集和分析

将所有患者肝脏 MRI 检查结果以 dcm 格式保存在本地硬盘，然后将上述数据输入 MIPAV（Medical Image Processing, Analysis, and Visualization）软件。采用画感兴趣区的方法画出每一层面的肿瘤残活以及坏死区域，通过计算机软件得出肿瘤强化区域和非强化区域的体积。肿瘤整体体积即肿瘤残活及坏死区域体积的总和。

10.2.2 统计分析

所有统计分析将采用 *Graphpad Prism* 统计软件。

10.2.3 资料的保存

研究者应当使资料保存完整，随时可取。

11 不良事件呈报

以下在 TACE/TAE 术中及术后可能出现的不良事件将会被监测：术中的血压、氧饱和度；术后疼痛、发热、肝功能变化；所有不良事件将会被记录。

12 临床试验进度

预计试验开始时间：2017 年 11 月

预计试验结束时间：2023 年 12 月
