# Supplementary material for: Bicarbonate-integrated transarterial chemoembolization (TACE) in real-world hepatocellular carcinoma
Source: Signal Transduct Target Ther. 2025 Sep 1;10:281. doi: 10.1038/s41392-025-02400-x (PMC12399753; doi:10.1038/s41392-025-02400-x)
Supplement: Supplementary file 2 — Supplementary file [file 41392_2025_2400_MOESM2_ESM.docx]

Supplementary Materials for

Bicarbonate-integrated TACE in real-world hepatocellular carcinoma

Kai Jin, Siying Zeng, Bin Li, Guangqiang Zhang, Jianjun Wu, Xun Hu, Ming Chao

Correspondence to: chaoming@zju.edu.cn, huxun@zju.edu.cn

**This PDF file includes:**

Materials and Methods

Figures. S1 to S3

Tables S1 to S8

Materials and Methods

The criteria for TILA-TACE treatment

1. No standard number of treatments (treatment as needed). Generally, an enhanced MRI scan, tumor-related markers, liver and kidney function tests, and routine blood tests are re-examined 4-6 weeks after the first TILA-TACE treatment. If imaging follow-up shows necrosis of the tumor tissue without enhancement and no new lesions, TILA-TACE treatment may not be necessary for the time being. The need for further TACE treatment and its frequency should be determined based on follow-up results, primarily considering the patient's response to the previous treatment, changes in liver function, and physical condition. The number of TILA-TACE treatments received by each patient is recorded.
2. The interval between treatment is about 1-3 months.
3. Treatment indications: Treatment indications are according to the Guidelines for the Diagnosis and Treatment of Primary Liver Cancer in China.

4. Stopping criteria:

*Termination by investigators*

(1)Severe liver dysfunction (Child-Pugh class C), including jaundice, hepatic encephalopathy, refractory ascites, or hepatorenal syndrome;

(2)Severe coagulation dysfunction that cannot be corrected;

(3)Completely embolized main portal vein with few collateral blood vessels formed;

(4)Active HBV infection or active infections that complicate the condition and cannot be treated simultaneously;

(5)Wide distant metastasis with an estimated survival of <3 months;

(6)Cachexia or multiple organ failure;

(7)Liver tumors accounting for ≥70% of the entire liver (staged embolization with small amounts of iodized oil emulsion can be considered if liver function is normal);

(8)Significant reductions in peripheral blood leukocytes and platelets, white blood cell (WBC) count <3.0 × 10^9^/L (when due to hypersplenism but not toxicity of chemotherapy; therefore, it is not an absolute contraindication for TACE), and platelets <50 × 10^9^/L;

(9)Renal dysfunction (creatinine >2 mg/dL or creatinine clearance rate <30 mL/min).

*Termination by patients*

The patient has no desire for treatment.

5. Follow up plan: The follow-up interval is 1-3 months, during which MRI scans are used to assess the viability of tumors, along with tests for tumor-related markers, liver and kidney function, and routine blood tests. These assessments determine whether further treatment is necessary.

6. The response of the target tumor to treatment was assessed 30 days after the first treatment and in the follow up visit with MRI, including the final follow-up.

Safety assessment system

1. The adverse events were scored according to Common Terminology Criteria for Adverse Events version 5.0
2. Safety endpoints

(1)Severe liver dysfunction (Child-Pugh class C), including jaundice, hepatic encephalopathy, refractory ascites, or hepatorenal syndrome;

(2)Severe coagulation dysfunction that cannot be corrected;

(3)Completely embolized main portal vein with few collateral blood vessels formed;

(4)Active HBV infection or active infections that complicate the condition and cannot be treated simultaneously;

(5)Wide distant metastasis with an estimated survival of <3 months;

(6)Cachexia or multiple organ failure;

(7)Liver tumors accounting for ≥70% of the entire liver (staged embolization with small amounts of iodized oil emulsion can be considered if liver function is normal);

(8)Significant reductions in peripheral blood leukocytes and platelets, white blood cell (WBC) count <3.0 × 10^9^/L (when due to hypersplenism but not toxicity of chemotherapy; therefore, it is not an absolute contraindication for TACE), and platelets <50 × 10^9^/L;

Renal dysfunction (creatinine >2 mg/dL or creatinine clearance rate <30 mL/min).

The planned intervention measures of the adverse events

1. Pain: pain relief medications;
2. Fever: antipyretic drugs, such as non-steroidal drugs;
3. Nausea and vomiting: antiemetic drugs;
4. Hepatic insufficiency, liver failure, hepatic encephalopathy: treatment with hepatoprotective drugs, blood ammonia-lowering drugs (such as ornithine aspartate, lactulose), diuresis and ascites paracentesis;
5. Infection (biloma, liver abscess): antibiotics, drainage;
6. Peptic ulcer: gastroprotective drugs such as PPI;
7. Gastrointestinal bleeding: hemostatic drugs, somatostatin, gastroprotective drugs such as PPI, surgical intervention, interventional embolization of bleeding arteries;
8. Renal insufficiency: diuresis, renal replacement therapy such as dialysis;
9. Myelosuppression: colony-stimulating factor, erythropoietin, blood transfusion;
10. Implantation metastasis via puncture path: Treatment of metastatic lesions such as radiotherapy;
11. Pulmonary embolism: symptomatic treatment such as oxygen inhalation, severe cases require surgical intervention;
12. Complications of endovascular procedures (puncture site hematoma, pseudoaneurysm, arterial dissection): observation, endovascular treatment such as arterial stent, surgical treatment, etc.

Figure. S1.


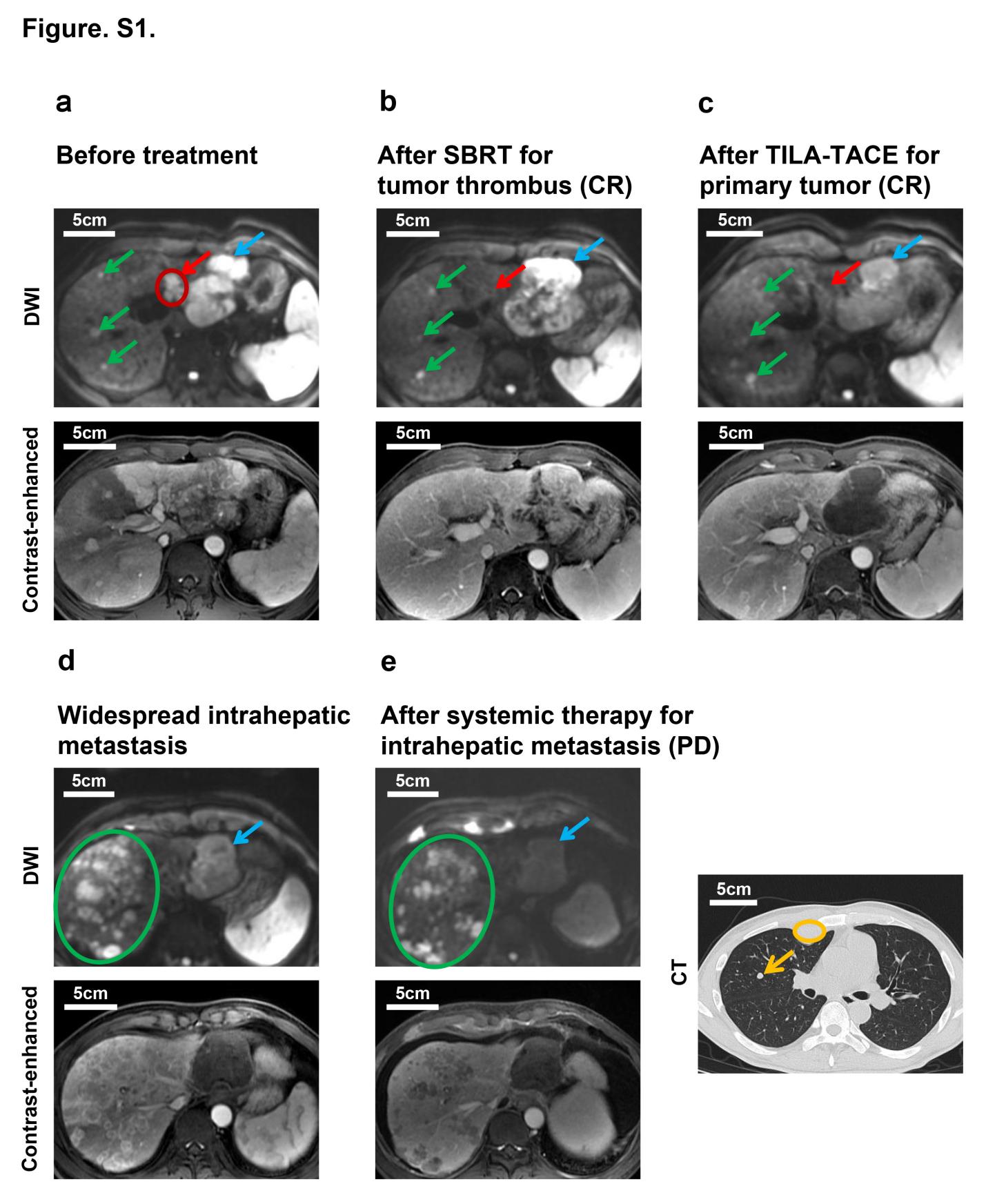


**Figure. S1. Representative MRI and CT scans of a patient who received SBRT, TILA-TACE, and systemic therapy.** (a) The patient had primary tumor (blue arrow), tumor thrombus at left portal vein branch (red arrow) and intrahepatic metastasis (green arrow) before treatment. (b) The SBRT radiation field was the red circle area in (a). 20 days after SBRT treatment, the tumor thrombus was cleared, while the primary tumor was significantly enlarged in comparison to that in (a). (c) Then the primary tumor was treated with TILA-TACE. A month after TILA-TACE treatment, the primary tumor was cleared. (d) Four months later, the targeted primary tumor remained in CR, continuing to shrink and absorb further. However, widespread intrahepatic metastasis had developed (green circle). The patients then received systemic therapy. (e) Two months after systemic therapy (targeted therapy and immunotherapy), the widespread intrahepatic metastasis showed a partial response to treatment. However, new extrahepatic metastases developed in the lung (yellow arrow) and chest wall (yellow circle).

Figure. S2.


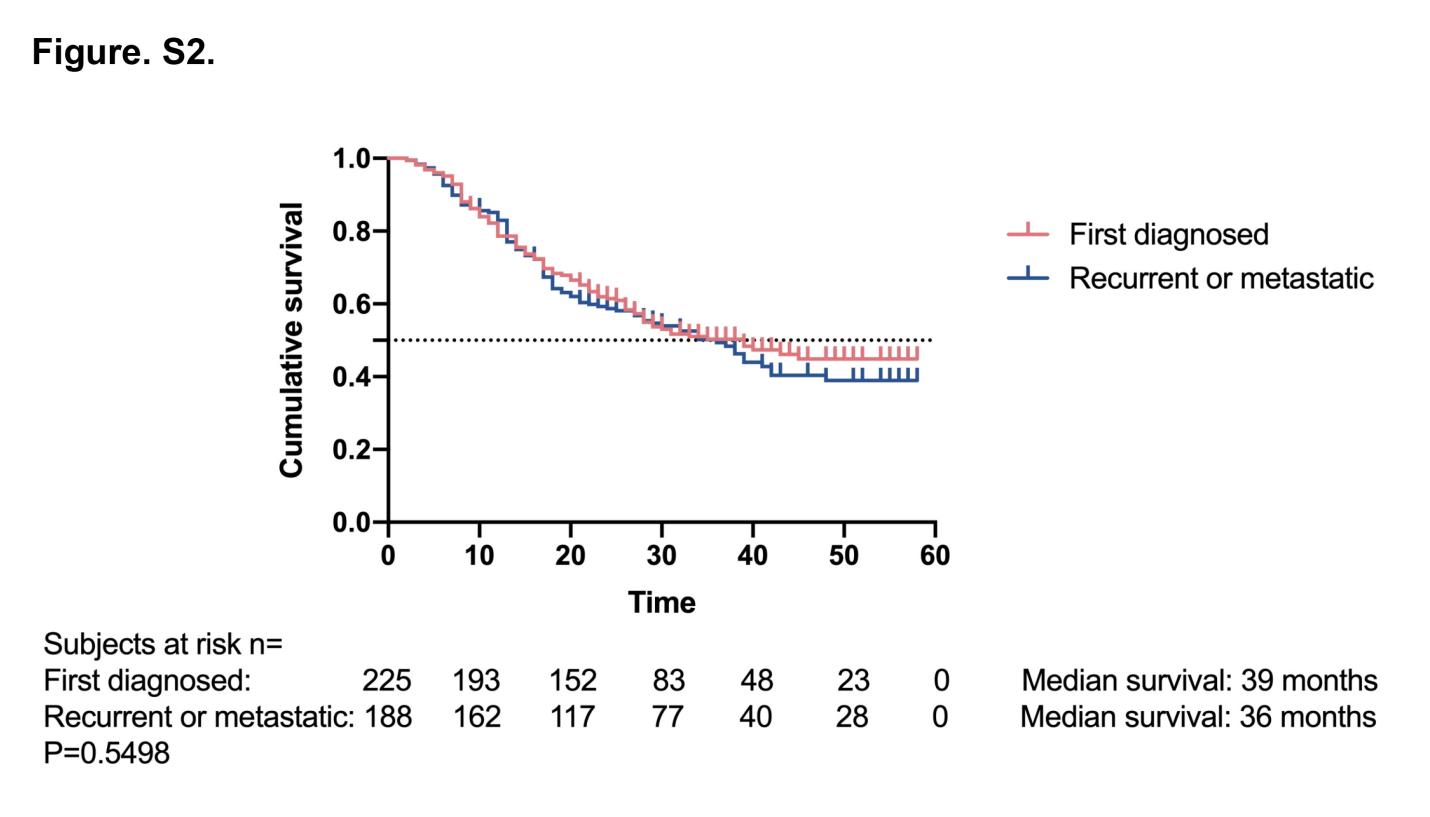


**Figure. S2. Kaplan-Meier analysis of cumulative survival of patients with newly diagnosed, recurrent, or metastatic disease.**

Figure. S3.


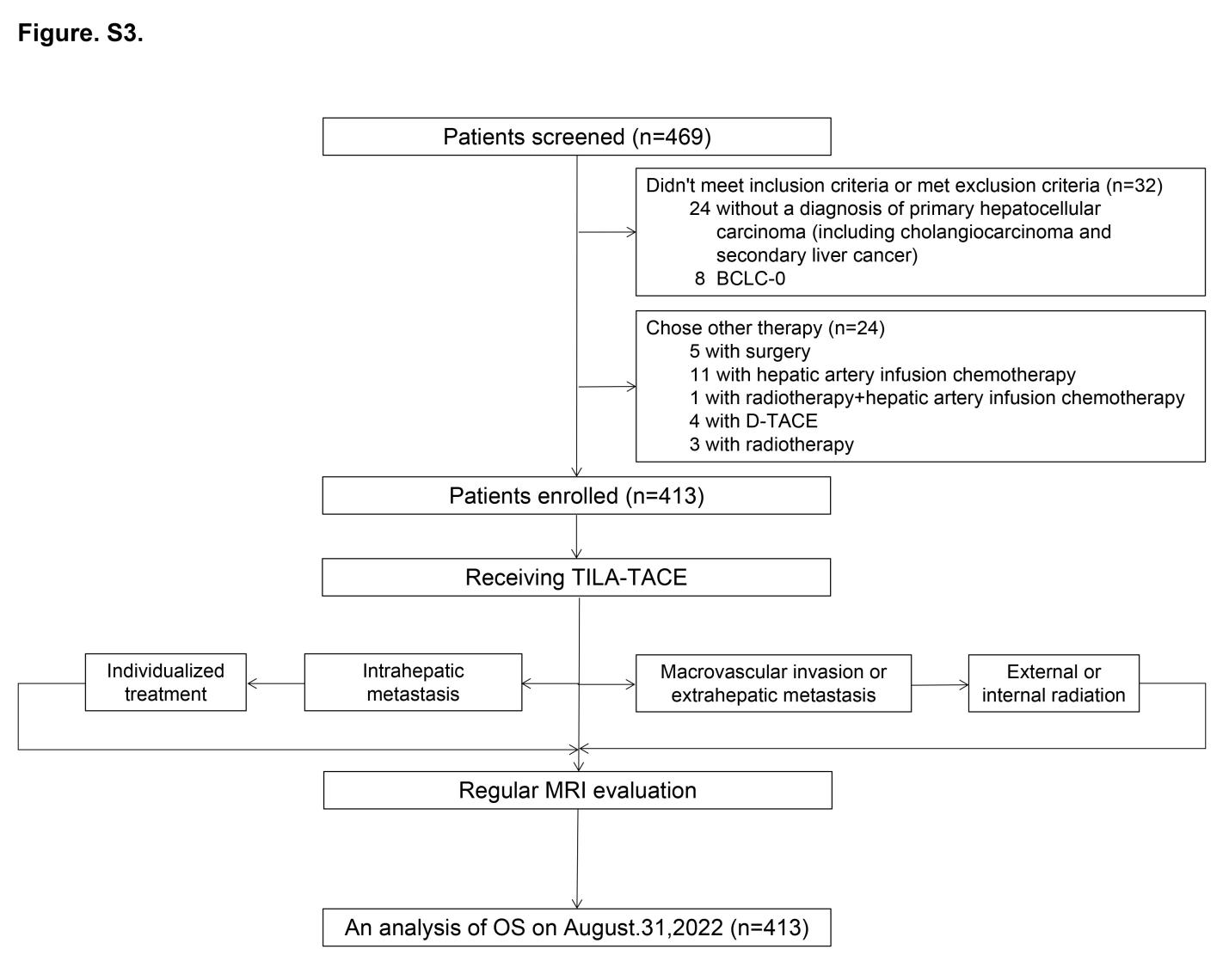


**Figure. S3. Trial flow diagram.**

Table S1.

|  | **Patients** |  |
| --- | --- | --- |
| **Efficacy estimation after the first round of TILA-TACE treatment** | **413** |  |
| CR | 152 | (36.80%) |
| PR | 254 | (61.50%) |
| SD | 3 | (0.73%) |
| PD | 4 | (0.97%) |
| ORR | 98.31% |  |

Table S1. The objective response rate after a single round of TILA-TACE treatment.

Table S2.

| **Rounds of TILA-TACE** | **Patients number** | **Percentage (%)** |
| --- | --- | --- |
| 1 | 179 | 43.34 |
| 2 | 131 | 31.72 |
| 3 | 52 | 12.59 |
| >3 | 51 | 12.35 |

Table S2. The number of rounds the patient received TILA-TACE.

Table S3.

|  | **Patients** |  | |
| --- | --- | --- | --- |
| **Efficacy estimation after multiple rounds of TILA-TACE treatment** | **404*** |  | |
| CR | 294 | | (72.77%) |
| PR | 106 | | (26.24%) |
| SD | 2 | | (0.50%) |
| PD | 2 | | (0.50%) |
| ORR | 99.01% | |  |
| **Fail to comply with multiple rounds of treatment** | **6** | |  |
| **Fail to estimate without imaging examinations** | **3** | |  |
| * Nine patients who failed to comply with multiple rounds of treatment and were not evaluable without imaging examinations were excluded. | | | |

Table S3. The objective response rate after multiple rounds of TILA-TACE treatment.

Table S4.

|  | **Patients** |  |
| --- | --- | --- |
| **Macrovascular invasion** | **270** |  |
| **With vascular tumor thrombus** | **132** |  |
| *Efficiency estimation* | *128* |  |
| CR | 122 | (95.31%) |
| PR | 2 | (1.56%) |
| SD | 0 | (0%) |
| PD | 4 | (3.13%) |
| **ORR** | **96.88%** |  |
| *Fail to estimate without imaging examinations* | *3* |  |
| *Didn’t receive treatment* | *1* |  |
| **Without vascular tumor thrombus** | **138** |  |
| *Efficiency estimation* | *133* |  |
| No tumor thrombus formation after treatment | 130 | (97.74%) |
| Tumor thrombus formation | 3 | (2.26%) |
| *Fail to estimate without imaging examinations* | *2* |  |
| *Didn’t receive treatment* | *3* |  |

Table S4. The efficacy estimation of macrovascular invasion.

Table S5.

|  | **Patients** |  |
| --- | --- | --- |
| **The treatment of extrahepatic metastasis ^*^** | **35** |  |
| *Efficacy estimation* | *33* |  |
| CR | 13 | (39.39%) |
| PR | 10 | (30.30%) |
| SD | 1 | (3.03%) |
| PD | 9 | (27.27%) |
| **ORR** | **69.70%** |  |
| *Fail to estimate without imaging examinations* | *2* |  |
| *, not all the patients with extrahepatic metastasis received treatment, considering physical state, economic status，or some other factors(The number of HCC patients with EHS is 60, and 35 received extrahepatic metastasis treatment) | | |

Table S5. The efficacy estimation of extrahepatic metastasis.

Table S6.

|  | **Preoperative** | **Postoperative of first**  **round TILA-TACE** | **Recovery after first**  **round TILA-TACE treatment** | **Recovery after multiple**  **rounds TILA-TACE treatment** |
| --- | --- | --- | --- | --- |
| **Child-Pugh class** |  |  |  |  |
| A | 365 | 336 | 389 | 380 |
| B | 48 | 76 | 24 | 33 |
| C | 0 | 1 | 0 | 0 |
| **Liver enzyme level** |  |  |  |  |
| ALT (U/L) | 42.14 ± 46.69 | 259.73 ± 433.67 | 40.75 ± 187.27 | 32.93 ± 26.47 |
| AST (U/L) | 55.85 ± 51.99 | 393.23 ± 815.34 | 42.13 ± 37.60 | 45.60 ± 38.73 |
| **Bilirubin (μM)** | 18.07 ± 9.56 | 28.13 ± 14.05 | 17.54 ± 9.12 | 20.82 ± 31.10 |

The biochemical tests of ALT, AST, and bilirubin were done within 1 or 2 weeks post operation.

Table S6. Liver function and biochemical indexes before and after treatment.

Table S7.

|  | **CRR** | | | **Logistic regression** | | |
| --- | --- | --- | --- | --- | --- | --- |
| **Subgroup category** | **No.** | **CRR** | **(95% CI)** | **OR** | **(95% CI)** | **p value** |
|  | **413** | **0.368** | **0.321-0.417** |  |  |  |
| **Prior TILA-TACE** |  |  |  |  |  |  |
| First diagnosed | 225 | 0.373 | 0.310-0.440 | 1.051 | 0.703-1.571 | 0.807 |
| Recurrent or metastatic | 188 | 0.362 | 0.293-0.435 | Ref |  |  |
| **Aetiology** |  |  |  |  |  |  |
| HBV | 349 | 0.367 | 0.316-0.420 | 0.965 | 0.556-1.675 | 0.900 |
| HCV | 0 | 0 |  |  |  |  |
| Non B-non C | 64 | 0.375 | 0.257-0.505 | Ref |  |  |
| **AFP** |  |  |  |  |  |  |
| ≤20 ng/mL | 161 | 0.416 | 0.339-0.496 | 1.978 | 1.226-3.191 | 0.005 |
| >20, ≤400 ng/mL | 101 | 0.446 | 0.347-0.548 | 2.230 | 1.308-3.802 | 0.003 |
| >400 ng/mL | 151 | 0.265 | 0.196-0.343 | Ref |  |  |
| **Child-Pugh class** |  |  |  |  |  |  |
| A | 365 | 0.381 | 0.331-0.433 | 1.656 | 0.847-3.238 | 0.141 |
| B | 48 | 0.271 | 0.153-0.418 | Ref |  |  |
| **ECOG (score)** |  |  |  |  |  |  |
| 0 | 69 | 0.725 | 0.604-0.825 | 6.244 | 3.507-11.114 | <0.001 |
| 1 | 344 | 0.297 | 0.249-0.348 | Ref |  |  |
| **The size of largest tumor** |  |  |  |  |  |  |
| >10 cm | 76 | 0.053 | 0.015-0.129 | Ref |  |  |
| >5 cm, ≤10 cm | 144 | 0.333 | 0.257-0.417 | 9.000 | 3.103-26.102 | <0.001 |
| ≤5 cm | 193 | 0.518 | 0.445-0.590 | 19.355 | 6.802-55.071 | <0.001 |
| **CNLC stage** |  |  |  |  |  |  |
| I | 40 | 0.800 | 0.644-0.909 | 9.490 | 4.228-21.302 | <0.001 |
| II | 29 | 0.621 | 0.423-0.793 | 3.882 | 1.771-8.511 | 0.001 |
| III | 344 | 0.297 | 0.249-0.348 | Ref |  |  |

Table S7. Subgroup analysis of CRR after first round TILA-TACE treatment.

Table S8.

|  | **CRR** | | | **Logistic regression** | | |
| --- | --- | --- | --- | --- | --- | --- |
| **Subgroup category** | **No.** | **CRR** | **(95% CI)** | **OR** | **(95% CI)** | **p value** |
|  | **404** | **0.728** | **0.682-0.771** |  |  |  |
| **Prior TILA-TACE** |  |  |  |  |  |  |
| First diagnosed | 220 | 0.823 | 0.766-0.871 | 2.916 | 1.848-4.600 | <0.001 |
| Recurrent or metastatic | 184 | 0.614 | 0.540-0.685 | Ref |  |  |
| **Aetiology** |  |  |  |  |  |  |
| HBV | 341 | 0.721 | 0.670-0.768 | 0.809 | 0.433-1.514 | 0.508 |
| HCV | 0 |  |  |  |  |  |
| Non B-non C | 63 | 0.762 | 0.638-0.860 | Ref |  |  |
| **AFP** |  |  |  |  |  |  |
| ≤20 ng/mL | 157 | 0.764 | 0.690-0.828 | 1.403 | 0.844-2.332 | 0.191 |
| >20, ≤400 ng/mL | 98 | 0.714 | 0.614-0.801 | 1.082 | 0.617-1.895 | 0.784 |
| >400 ng/mL | 149 | 0.698 | 0.617-0.770 | Ref |  |  |
| **Child-Pugh class** |  |  |  |  |  |  |
| A | 359 | 0.744 | 0.695-0.788 | 1.935 | 1.018-3.676 | 0.044 |
| B | 45 | 0.600 | 0.443-0.743 | Ref |  |  |
| **ECOG (score)** |  |  |  |  |  |  |
| 0 | 68 | 0.956 | 0.876-0.991 | 10.124 | 3.111-32.944 | <0.001 |
| 1 | 336 | 0.682 | 0.629-0.731 | Ref |  |  |
| **The size of largest tumor** |  |  |  |  |  |  |
| >10 cm | 75 | 0.587 | 0.467-0.699 | Ref |  |  |
| >5 cm, ≤10 cm | 137 | 0.825 | 0.751-0.884 | 3.317 | 1.755-6.270 | <0.001 |
| ≤5 cm | 192 | 0.714 | 0.644-0.776 | 1.755 | 1.006-3.060 | 0.047 |
| **CNLC stage** |  |  |  |  |  |  |
| I | 40 | 0.975 | 0.868-0.999 | 18.223 | 2.471-134.401 | 0.004 |
| II | 28 | 0.929 | 0.765-0.991 | 6.074 | 1.416-26.062 | 0.015 |
| III | 336 | 0.682 | 0.629-0.731 | Ref |  |  |

Table S8. Subgroup analysis of CRR after multiple rounds TILA-TACE treatment.
